# Malaria and associated factors among under-five children in Borena pastoral communities, southern Ethiopia

**DOI:** 10.3389/fpara.2024.1438218

**Published:** 2024-08-01

**Authors:** Alqeer Aliyo, Wako Golicha, Anteneh Fikrie

**Affiliations:** ^1^ Medical Laboratory Science Department, Institute of Health, Bule Hora University, Bule Hora, Ethiopia; ^2^ School of Public Health, Institute of Health, Bule Hora University, Bule Hora, Ethiopia

**Keywords:** malaria, under-five children, pastoral communities, Borena, southern Ethiopia

## Abstract

**Background:**

Malaria continues to be an important threat to public health and infects millions of children under 5 years of age each year. Although Ethiopia has set targets for at-risk group interventions to eradicate and manage malaria, the illness is still a serious public health problem in areas where it is endemic, especially in the unique lowlands in the Borena zone.

**Objective:**

This study aimed to determine the prevalence of malaria and associated factors among children in Borena’s pastoral communities, Oromia Regional State, southern Ethiopia, in 2022.

**Methods:**

A community-based cross-sectional study was conducted from 1 March to 30 April 2022 among 437 randomly selected households with children under 5 years of age in pastoral communities in the Borena zone. Data were collected through face-to-face interviews with structured and pretested questionnaires and blood sample examination using microscopy. Thick and thin blood smears were prepared and examined under a microscope at a health center to confirm malaria cases. The data were analyzed using SPSS version 25. Bivariate and multivariable logistic regression analyses were used to identify factors associated with malaria, and a *p*-value <0.05 was used to declare statistical significance.

**Result:**

The prevalence of malaria among children under 5 years of age was 27.8% (95% CI = 23.5–32.1), and the prevalence rates of *Plasmodium falciparum*, *Plasmodium vivax*, and mixed malaria were 68.4%, 25.6%, and 6%, respectively. Regarding the proportion of malaria among age groups, 81% of children under 5 years of age between 48 and 59 months were malaria-positive. In this study, fever within the last week (AOR = 13.34, 95% CI = 6.37–27.95) and not sleeping under insecticide-treated nets (ITNs) (AOR = 3.10, 95% CI =1.95–4.92) were significantly associated with malaria. The age of the children was negatively associated with malaria prevalence.

**Conclusion:**

The prevalence of malaria among children under 5 years old was high during the rainy season in this pastoral region of Ethiopia. Factors such as fever within the last week and not sleeping in insecticide-treated nets were significantly associated with malaria. Therefore, to reduce malaria-related infections and deaths among children under 5 years of age, the government ought to enhance the availability and utilization of insecticide-treated nets (ITNs).

## Introduction

Malaria, caused by the *Plasmodium* species, is one of the biggest threats to public health and can be fatal. Globally, approximately 241 million malaria cases and 627,000 malaria deaths occurred in 2020, with an estimated increase of 14 million cases and 69,000 deaths in 2019. Children under 5 years of age account for the vast majority of malaria mortality in sub-Saharan Africa (World Malaria Report, 2020).

Malaria is still a major challenge for public health and socioeconomic development. In particular, in underdeveloped countries, malaria is one of the leading causes of mortality among pregnant women and children under 5 years of age, especially in sub-Saharan Africa, including Ethiopia (Deressa et al., 2005). Some studies conducted in the region reported different prevalence rates of malaria among children under 5 years of age: 12.2% in Rwanda’s Huye districts (Nyirakanani et al., 2018), 41.4% in Ghana’s Hohoe lowland attitudes (Kweku et al., 2017), and 19.7% in Uganda (Roberts and Matthews, 2016).

Malaria is a major infection that affects resource-constrained countries, particularly in tropical regions around the world. Ethiopia is one of the countries at high risk for malaria epidemics due to changes in climate and topography that influence both vector and parasite populations. Climatic fluctuations and drought-related nutritional emergencies cause a wide range of epidemics to occur every 5 to 8 years in some areas. *Plasmodium falciparum* and *P. vivax* are the two predominant species in the country, transmitted by the inoculation of mosquitoes (*Anopheles* species, including *Anopheles arabiensis*) (Fontaine et al., 1961; Carter, 2012; PMI, 2012; FMOH, 2015). *Plasmodium falciparum*, *P. vivax*, and mixed infections account for an estimated 65%, 34%, and 1% of malaria cases, respectively (Federal Ministry of Health Ethiopia, 2020).

In Ethiopia, three-fourths of the areas are endemic to malaria and more than two-thirds of its population (more than 55 million people) live in areas at risk for malaria. Nevertheless, the distribution of malaria species differs across geographical regions, and the associated risks have not been updated to reflect recent changes such as urbanization and land use modifications. The incidence of malaria cases was 28 per 1,000 people at risk in 2020, and the mortality rate of children under 5 years of age per 1,000 live births was 59 in 2019 (U.S. President’s Malaria Initiative Ethiopia Malaria Operational Plan FY 2022; Adugna, 2007; Haji et al., 2016; Tadesse. et al., 2018).

According to the Ethiopian Federal Ministry of Health (FMoH) estimation, approximately 5–10 million clinical malaria cases and 70,000 deaths occur due to malaria each year. Half of those cases and deaths were caused by *P. falciparum*. Major malaria epidemics occur every 5 to 8 years (FMoH, 2012). Recently, cross-sectional studies conducted in different ecogeographic regions of the country in children under the age of 5 reported malaria prevalence rates from Oromia East Shoa (20.5%), Afar Dubti district (64%), and Arba Minch Zuria district (22.1%). Factors such as net use and availability, family history of malaria, and mosquito breeding sites near houses were associated with malaria (Tadesse. et al., 2018; Woday et al., 2019; Abossie et al., 2020).

The impact of malaria also extends beyond the health center to families and everyday lives: children may develop long-term neurological sequelae following severe malaria attacks and further subtle developmental and cognitive impairments as a result of both severe and uncomplicated episodes, and families may also face substantial economic consequences. It causes severe anemia, acute renal failure, hypoglycemia, and severe complications (Holding and Kitsao-Wekulo, 2004). Although malaria can affect all populations, children under 5 years old are the most vulnerable group affected by the disease, accounting for 61% of all malaria deaths worldwide (World Health organization(WHO), 2018). Some other population groups, including pregnant women and patients with HIV/AIDS, as well as non-immune migrants, mobile populations, and travelers, have a considerably higher risk of contracting malaria and developing severe disease (FMOH, 2006).

In Ethiopia, a program is being carried out to eradicate malaria by 2030. The prevalence and determinants of malaria among vulnerable groups should be examined over time and in various locations to gauge the success (Federal Ministry of Health Ethiopia, 2020). Currently, rapid diagnostic tests (RDTs) are used to diagnose malaria and treat patients. Indoor residual spraying (IRS) with insecticides, promotion of community ownership, and long-lasting insecticide nets (LLINs) are methods that have been scaled up to increase access to malaria prevention and control (Jakubowski et al., 2017). This has numerous difficulties because it is not grounded in local circumstances, and human migration can swiftly derail attempts to stifle or stop transmission. Additionally, there is a lack of reporting on community-level active case detection and widespread screening in the nation’s intervention policies and strategies.

In addition, knowing the current prevalence of malaria among children under 5 years of age in pastoralist communities in Ethiopia and describing the factors associated with the prevalence of malaria are of paramount importance to scaling up and designing appropriate interventions for the remotest southern Ethiopian pastoralists, who are vulnerable due to their mobility nature. Nevertheless, there has been no prior research on the prevalence of malaria among children under 5 years old and the potential factors associated with it in Borena pastoral communities. Therefore, the present study aimed to assess the prevalence of malaria and associated factors among children under 5 years of age in pastoral communities in Borena, South Ethiopia.

## Materials and methods

### Study setting and period

The community-based cross-sectional study was conducted on pastoral communities of the Borena zone, Oromia Regional State, South Ethiopia, from 1 March to 30 April 2022. The Borena zone has 10 districts and is bordered by the Somali region to the east, the west Guji region to the north, northeast Kenya to the south, and the SNNP region to the west. The Borena zone is one of the hotspot areas within the region. According to the Central Statistics Agency for 2019, the zone population is estimated at approximately 503,877. Approximately 89% of the population lives in 134 rural pastoralist kebeles in the zone. The altitude of the zone is 1,500 m below sea level. The mean annual temperature ranges from 27°C to 29°C. The areas of the Borena zone are known to be malaria-endemic, but transmission is seasonal. Peak transmission is usually reached between September and November, shortly after the large and small rainy seasons, respectively. Regarding health facilities, only 1 general hospital, 4 primary hospitals, and 44 health centers were found in the zone (22).

### Study population and selection

The study included all randomly selected children under 5 years old from selected households in kebeles across three districts of the Borena zone during the study period, while children of households who recently immigrated and not registered in the kebele administration and children who had received antimalarial chemotherapy 42 days before the gathering of data were excluded from the study.

### Sample size determination

The minimum required sample size of this study was determined using a single population proportion based on a prevalence rate (*p* = 22.1%) taken from a previous study conducted in Arba Minch (Abossie et al., 2020), with the assumption of precision or degree of error of 0.05, confidence of interval 95%, and non-response rate of 10%.


$$n=\frac{{\text{Z}}^{2}\alpha /2\text{P}(1-\text{p})}{{\text{d}}^{2}}=\frac{{\left(1.96\right)}^{2}(0.221)(1-0.221)}{0.05^{2}}=264.5\approx 265$$

We added a 10% non-response rate and considered a 1.5 design effect. Then, the final sample size became [265 + (0.1 * 265)] * 1.5 = 437.25. The final sample size was 437 children under 5 years old in selected households of the kebeles in three districts of the Borena zone.

### Sampling technique and procedure

**Figure 1:**
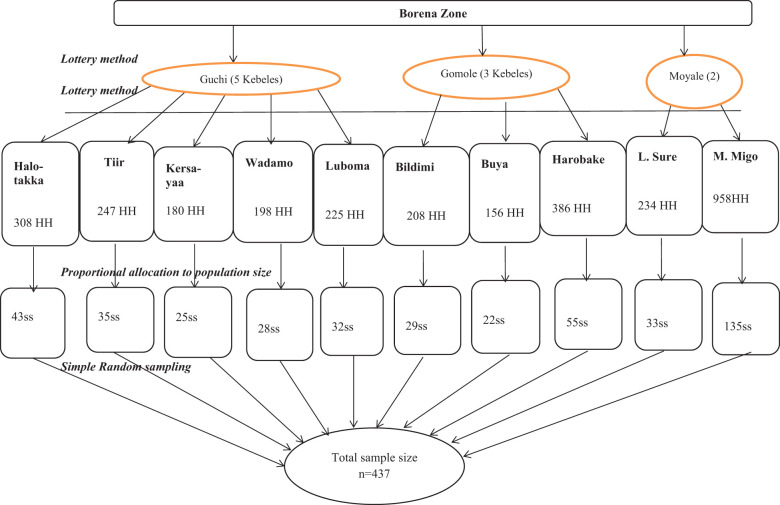
Sampling procedure of the prevalence of malaria and associated factors among children under 5 years old from pastoral communities in the Borena zone. HH, households; ss, sample size (allocated sample size for each kebele); *n*, total sample size.

Multistage sampling procedures were used, followed by probability proportional allocation to determine sample size. In the first stage, three districts (Guchi, Gomole, and Moyale) were randomly selected from 10 districts in the Borena zone. In the second stage, 10 kebeles were selected using the lottery method: 5 of 18 eligible kebeles from Guchi, 3 of 13 eligible kebeles from Gomole, and 2 of 6 eligible kebeles from Moyale. Based on the number of households in each kebele, proportional population size allocation (PPS) was used to allocate the calculated sample size to each kebele. The study population was children under 5 years old in selected households, and their parents or guardians were residents of pastoral communities in randomly selected kebeles in Guchi, Gomole, and Moyale districts. An updated kebele-based sampling frame of households that had under-five children was obtained from the community health workers/kebele administration office. There are 3,100 households with children under 5 years old registered in 10 selected kebeles. A simple random sampling method was applied within each kebele to select 437 households (**Figure 1**). Participants in selected households were visited at their homes, informed about the study, and invited to participate. If the participant was not at home, the research teams visited the house a minimum of three times. An eligible child was selected from each household using the lottery method in cases where more than one child under 5 years of age lived in the house (Fikrie et al., 2021).

### Data collection methods

#### Data collection tools and procedures

The data were collected using pretested standard questionnaires. Data collectors introduced themselves and explained the objective of the study, highlighting the benefit of being tested for malaria. Additionally, data collectors gave details to the child’s parents/caregivers/guardians so that no names of the participants were written on the questionnaire, confidentiality was protected, and written consent was obtained. Data were collected through face-to-face interviews with the children’s mothers/guardians. Then, a questionnaire was completed to obtain demographic information and associated factors for malaria infection, such as family history of malaria and indoor residual spraying, and if children slept under insecticide-treated mosquito nets.

#### Blood sample collection and testing

After the interview was completed, 1–2 µL of blood volumes were obtained through a finger prick using sterile lancets and capillary tubes from each child included in the sampling, regardless of the signs and symptoms of malaria. Thick and thin smears were prepared. The slides were labeled with participant codes, packed into a slide bag after being air-dried, and then transported to a nearby health center for microscope examination. Trained laboratory technicians examined properly fixed 10% Giemsa-stained smears under ×100 microscopes for the presence of malaria parasites according to the National Malaria Diagnosis Guideline (NMG) (see **Supplementary File 1**). The presence of parasites and species was determined from the thick and thin smears, respectively (Sharew et al., 2009). If one or more asexual stages of the *Plasmodium* (trophozoite, ring stage, merozoite, and/or gametocyte) were present, it was taken as a positive result. A minimum of 100 consecutive fields were counted in the thick blood film before a slide was classified as negative (Cheesbrough, 2008). Five percent of the slides were randomly selected and then blindly checked for consistency by an experienced medical laboratory technologist.

### Operational definition and definitions of terms

*Malaria* is a mosquito-borne disease caused by the *Plasmodium* species. People with malaria often experience fever, chills, and flu-like illnesses (Aychiluhm et al., 2020).

*Kebeles* is the smallest administrative unit in Ethiopia (Aliyo et al., 2022).

Monthly household income was classified as follows: low = 1,000 ETB, medium = 1,001–2,575 ETB, and high >2,575 ETB (Fikrie et al., 2021).

### Data quality control

The data collectors were trained on the techniques of data collection for 2 days. The questionnaires were initially prepared in English and then translated into the Afan Oromo language. Then, the Afan Oromo language version was translated back into English to check for any inconsistencies or distortions in the meaning of words and concepts. Experts assessed the relevance of the questionnaires, and their comments were taken into consideration. Then, 5% of the questionnaires were pretested outside the study area, which was similar to the study population, before beginning the actual data collection process to assess the clarity, understandability, and flow of each question. Based on the results of the pretest, the questionnaire was modified before the actual data were collected. During data collection, questionnaires were checked each day at the actual data collection time for completeness and consistency. Standardized procedures were strictly followed during the blood sample collection, smear storage, transportation, and analytical processes. Positive and negative controls were followed according to the microscopy slide quality assurance and test manual.

### Data processing and analysis

All completed checklists were verified for completeness and consistency, and then double data entry was performed using EpiData version 3.5.1 software. Then, the data were exported to SPSS version 25.00 for further analysis. Descriptive statistics such as percentage and frequency were calculated, and the mean with standard deviation or median with interquartile range was used to summarize qualitative data. Different types of graphs, charts, and tables were used to present the data. Bivariate and multivariable logistic regression analyses were used to identify factors associated with the prevalence of malaria. Variables in the bivariate analysis with a *p*-value less than 0.25 (*p* < 0.25) were included in the multivariate analysis. Multivariable logistic regression was performed to control for potential confounders. The model’s fitness was checked using the Hosmer–Lemeshow goodness-of-fit test. Finally, the strength of associations between outcome and determinant variables was expressed using adjusted odds ratios (AORs) with 95% confidence intervals using the Wald method, and the significance of associations was declared at a *p*-value of less than 0.05.

### Ethical approval and consent to participate

The Institutional Review Board of Bule Hora University (BHU/IRB/2404/144) reviewed and approved the ethical clearance of the study. An official letter was written to the Guchi, Moyale, and Gomole districts. Informed voluntary consent was obtained in writing and signed by the child’s parents or primary guardian (aunt, uncle, grandmother, grandfather, etc.). Participation in the study was voluntary, and participants were informed of their right to quit/refuse their participation at any stage of the study if they did not want to participate. The codes were used to protect participant information, and neither the questionnaire nor the test tube contained any participant identifiers. Furthermore, the confidentiality of the information was ensured using an anonymous questionnaire. Participants were only interviewed alone to maintain privacy and free testing. Children who tested positive for malaria were referred to nearby health facilities for treatment.

## Results

### Sociodemographic characteristics

In this study, which included households with children under 5 years of age, 421 participated, yielding a 96.3% response rate. Children’s ages ranged from 6 to 59 months, with an average age of 22.6 months (SD ± 9.69). Of these participants, 219 (52%) were male children. The largest age group was children under 11 months, with a total number of 150 (35.6%), followed by those aged 12 to 23 months, totaling 128 (30.4%). A significant majority of mothers or guardians, 391 (92.9%), were illiterate, and 76 (18.1%) worked as herders. Most households, 345 (81.9%), had two or more children. Most of the children, 365 (86.7%), lived in rural areas. Only 64 households (15.2%) had a monthly income exceeding 2,575 Ethiopian birr.

### Mosquito prevention practices

The recent study revealed that 217 (52.5%) children did not use insecticide-treated nets (ITNs), and merely 5 (1.2%) households with children under 5 years of age had applied indoor residual spraying (IRS) in the last 1 year. The majority (*n* = 258, 61.3%) of homes had livestock. Of the children enrolled in the study, most (*n* = 269, 63.9%) had no fever history within the past week. In terms of household members, the majority (*n* = 283, 67.2%) were not infected with malaria in the last month.

### Prevalence of malaria among children under 5 years of age

**Figure 2:**
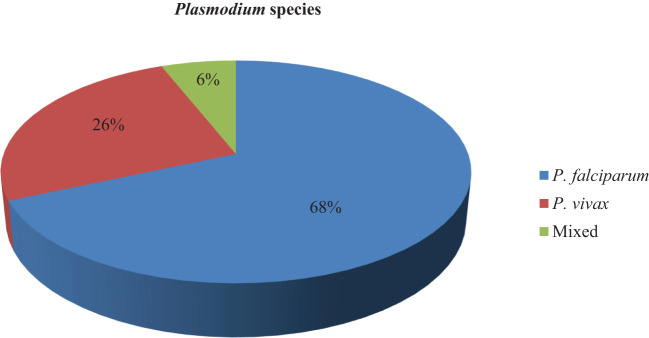
*Plasmodium* species diagnosed in children under 5 years of age.

The prevalence of malaria among children under 5 years old was 117 (27.8%) (95% CI = 23.5–32.1). There were two *Plasmodium* species diagnosed in children under 5 years old in households. Of the diagnosed species of malaria-causing agents, 80 (68.4%) (95% CI = 60–76.8) were *P. falciparum*, 30 (25.6%) (95% CI = 17.7–33.5) were *P. vivax*, and 7 (6%) (95% CI = 1.7–10.3) were mixed (**Figure 2**).

**Figure 3:**
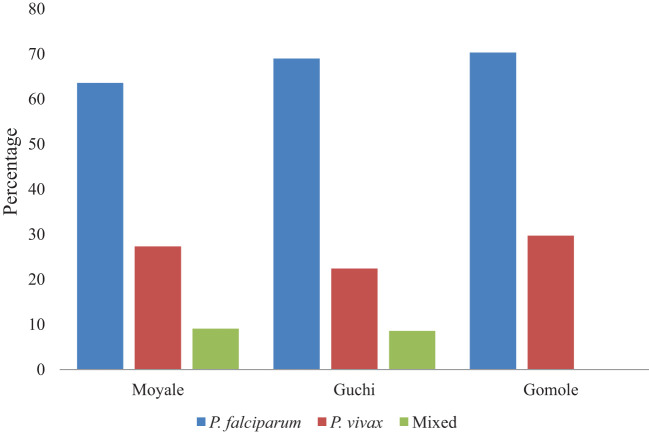
The proportion of children under 5 years old infected with *Plasmodium* species in selected districts.

*Plasmodium falciparum* was identified in 14 (63.6%) children under 5 years of age from the Moyale district, 40 (69%) in the Guchi district, and 26 (70.3%) in the Gomole district. *Plasmodium vivax* was found in 6 (27.3%) children from Moyale, 13 (22.4%) from Guchi, and 11 (29.7%) from Gomole districts. Mixed infections were detected in children from both the Moyale and Guchi districts. In terms of malaria distribution among districts, Guchi had the highest number of cases (30.2%), followed by Gomole with 37 cases (29.6%) and Moyale with 22 cases (21.2%) (**Figure 3**).

In terms of malaria within the age groups of children under 5 years of age, the malaria proportion was higher among children who were in the age group of 48–59 months (42, 80.8%), followed by children in the age groups of 24–35 months and 36–47 months (34, 70.8% and 21, 44.8%, respectively). *Plasmodium falciparum* was higher among the 24–35 and 48–59 age groups, with 27 (56.3%) and 28 (53.8%), respectively (**Table 1**).

**Table 1:** Prevalence of *Plasmodium* species within the age groups of children under 5 years of age, 2022.

Age groups	Frequency	*P. falciparum* (%)	*P. vivax* (%)	Mixed (%)	Total (%)
1–11	150	9 (6.0)	1 (0.7)	–	10 (6.7)
12–23	128	2 (1.6)	7 (5.5)	1 (0.8)	10 (7.9)
24–35	48	27 (56.3)	5 (10.4)	2 (4.2)	34 (70.8)
36–47	43	14 (32.6)	5 (11.6)	2 (4.7)	21 (48.8)
48–59	52	28 (53.8)	12 (23.1)	2 (3.8)	42 (80.8)

-, Not diagnosed.

### Factors associated with the prevalence of malaria among children under 5 years of age

In the bivariate analysis, child age groups, sleeping under ITN, and child fever history had a *p*-value of <0.25 and were considered candidates for multivariable analysis. Multivariable analysis showed the factors that were significantly associated with malaria among children under 5 years old. Children who were in the 24 to 35 months age group (AOR = 0.29, 95% CI = 0.09–0.89), those who were in the 36 to 47 months age group (AOR = 0.18, 95% CI = 0.06–0.52), those who were in the 12 to 23 months age group (AOR = 0.01, 95% CI = 0.00–0.03), and those who were in the 1 to 11 months age group (AOR = 0.01, 95% CI = 0.00–0.04) were less likely to have positive test results for malaria than those in the 48 to 59 months age group. Children who had a fever within the last week were 13 times more likely to be infected with malaria than those who did not have a fever (AOR = 13.34, 95% CI = 6.37–27.95). Children who did not sleep under ITN were three times more likely to be infected with malaria (AOR = 3.10, 95% CI = 1.95–4.92) (**Table 2**).

**Table 2:** Bivariate and multivariable analyses of factors associated with the prevalence of malaria among children under 5 years of age, 2022.

Variables	Category	Malaria		COR (CI)	AOR (CI)
		Positive (%)	Negative (%)		
Child’s age group (in months)	1–11	10 (6.7)	140 (93.3)	0.02 [0.01–0.04]***	0.01 [0.00–0.04]***
	12–23	10 (7.8)	118 (92.2)	0.02 [0.01–0.05]***	0.01 [0.00–0.03]***
	24–35	34 (70.8)	14 (29.2)	0.58 [0.23–1.46]*	0.29 [0.09–0.89]*
	36–47	21 (48.8)	22 (51.2)	0.23 [0.09–0.57]***	0.18 [0.06–0.52]**
	48–59	42 (80.8)	10 (19.2)	1	1
Child’s sex	Male	57 (53.3)	162 (46.7)	0.83 [0.54–1.28]	
	Female	60 (48.7)	142 (51.3)	1	
Mother/guardian’s educational status	Did not attend formal education	106 (90.6)	285 (93.8)	0.64 [0.30–1.40]	
	Attended formal education	11 (9.4)	19 (6.2)	1	
Mother/guardian’s occupation status	Herder	25 (32.9)	51 (67.1)	1	
	Housewife	56 (25.1)	167 (74.9)	0.68 [0.39–1.21]	
	Merchant	1 (4.3)	22 (95.7)	0.09 [0.01–0.73]	
	Farmer	22 (31.4)	48 (68.6)	0.94 [0.47–1.87]	
	Private employee	3 (30)	7 (70)	0.87 [0.21–3.67]	
	Government employee	5 (55.6)	4 (44.4)	2.55 [0.63–10.33]	
	Others	5 (50)	5 (50)	2.04 [0.54–7.70]	
Number of children in the household	One	22 (28.9)	54 (71.1)	1.07 [0.62–1.86]	
	Two and above	95 (27.5)	250 (72.5)	1	
Residence	Urban	14 (25)	42 (75)	0.85 [0.44–1.42]	
	Rural	103 (28.2)	262 (71.8)	1	
Income	Low ≤1,000 ETB	35 (29.9)	82 (27)	1.3 [0.64–2.55]	
	Medium = 1,001–2,575 ETB	66 (56.4)	174 (57.2)	1.14 [0.60–2.14]	
	High >2,575 ETB	16 (13.7)	48 (15.8)	1	
Child’s fever history within the past 1 week	Yes	87 (74.4)	65 (21.4)	10.66 [6.49–17.53]***	13.34 [6.37–27.95]***
	No	30 (25.6)	239 (78.6)	1	1
Family malaria history	Yes	41 (35)	97 (31.9)	1.15 [0.73–1.81]	
	No	76 (65)	207 (68.1)	1	
IRS in the house in the last 1 year	Yes	2 (40)	3 (60)	1	
	No	115 (27.6)	301 (72.4)	0.57 [0.10–3.47]	
Child sleeps under ITN	Yes	33 (16.5)	167 (83.5)	1	1
	No	84 (38)	137 (62)	3.10 [1.95–4.92]***	2.08 [1.06–4.10]*
Livestock present in the house	Yes	73 (28.3)	185 (71.7)	0.94 [0.60–1.45]	
	No	44 (27)	119 (73)	1	

COR, crude odds ratio; AOR, adjusted OR with precise 95% confidence interval.

Statistical significance at ***p < 0.001, **p < 0.01, and *p < 0.05.

## Discussion

The overall prevalence of malaria among children under 5 years of age during the rainy season in Borena pastoral communities, in southern Ethiopia, was 27.8% (95% CI = 23.5–32.1). The study’s finding was relatively in line with a study conducted in the Hadiya zone (25.8%) (Delil et al., 2016). However, the prevalence rate of the present study was lower compared to that in studies done in Ghana Hohoe municipality (41.4%) (Kweku et al., 2017); Dubti district, Afar region (64%) (Woday et al., 2019); and Kersa Woreda (41.8%) (Karunamoorthi and Bekelea, 2009). A possible reason for the discrepancy in prevalence may be that the children enrolled in the current study were children with or without malaria signs or symptoms, while other studies were conducted in malaria-suspected children who already developed signs or symptoms and that different laboratory examination techniques and technicians’ skills were employed.

However, the results of this study were higher than those of previous studies in Uganda (19.7%) (Roberts and Matthews, 2016); the Benishangul-Gumuz region (3.9%) (Ahmed et al., 2021); East Shewa zone (20.5%) (Tadesse. et al., 2018); Arba Minch Zuria district (22.1%) (Abossie et al., 2020); Woreta, northwestern Ethiopia (22.9%) (Alelign et al., 2018); and Yemen (9.8%) (Al-QuHaiti et al., 2022). The higher prevalence rate in this study could be attributed to the following: the study was conducted during the malaria transmission season, with pastoralists who moved to find pasture for their animals, which could spread disease throughout the community; there was a lack of awareness and preventative measures for treatments; and the study was carried out in a lowland malaria-endemic area.

The present study identified *Plasmodium* species that cause malaria infections among children under 5 years of age in the study area. Of the causative agents, the majority were caused by *P. falciparum* (68.4%), followed by *P. vivax* (25.6%) and mixed (6%). This finding is comparable with other studies conducted in the Ethiopia Dubti district, Afar region (66.5%) (Woday et al., 2019); Kersa Woreda (66%) (Karunamoorthi and Bekelea, 2009); and Woreta, northwestern Ethiopia (64.8%), with prevalence rates of 66.5%, 31.8%, and 3.4% for *P. falciparum*, *P. vivax*, and mixed infection, respectively (Alelign et al., 2018). Contrary to this finding, the prevalence of *P. falciparum* was higher than that in studies conducted in southern Rwanda (11.7%) (Gahutu et al., 2011); Huye district, southern Rwanda (12.2%) (Nyirakanani et al., 2018); Hadiya zone (25.5%) (Delil et al., 2016); Arba Minch Zuria district (50.0%) (Abossie et al., 2020); East Shewa zone (8.4%) (Tadesse. et al., 2018); and Kersa Woreda (33.3%) (Karunamoorthi and Bekelea, 2009). There are multiple reasons for *P. falciparum*’s greater dominance than *P. vivax*. These include the high rate of *P. falciparum*’s proliferation in the host cell, its ability to infect red blood cells of all ages, its resistance to first-line therapy, and the lowland climate of the study location, where *P. falciparum* is a common species.

Multivariate analysis indicated that children who did not sleep in ITN were nearly two times more likely to be infected with malaria than those who slept under ITN. This finding was supported by a study conducted in Arba Minch that reported that children who slept under ITNs were more likely to be protected from malaria infection than those who did not sleep under ITNs [17], in the Afar Dubti district (Woday et al., 2019) and in the Hadiya zone (Delil et al., 2016). However, this finding disagrees with a study conducted in Ghana that reported that ITN use did not have a significant association with malaria infection among children (Afoakwah et al., 2018). In the study area, the community uses ITN for kitchen sheltering, plant protection, thread making, selling to feed families, and other purposes. This may be the result of inadequate health education provided by health extension workers and other relevant organizations regarding malaria transmission prevention and control.

Children who had a history of fever within the last week were 13 times more likely to be infected with malaria than those who had not. This study is corroborated by the Ghana Hohoe Municipality (Kweku et al., 2017). Compared to those aged 48 to 59 months, children aged 24 to 35 months and 36 to 47 months were 71% and 82% less likely to have malaria, respectively, while those aged 12 to 23 months and 1 to 11 months were 99% less likely to have malaria-positive test results. This result is in agreement with previous studies conducted in the Hohoe municipality (Kweku et al., 2017) and Afar Dubti district (Woday et al., 2019). In addition, the prevalence of malaria among children aged 48 to 59 months was 80.8%, with *P. falciparum* species predominating. There could be three causes for this. First, children aged 48 to 59 months were more susceptible after the decline of maternally derived IgG as their own immunity begins to gradually build with repeated exposure to infection. Second, mothers typically slept with the youngest child in front of them and took great care of them; thus, these children were less likely to get bitten by mosquitoes than older children. Third, children between the ages of 48 and 59 months frequently play outside and have a higher risk of getting bitten by mosquitoes.

### Strengths and limitations

The strengths of the study were that data were gathered from randomly selected children under 5 years old and from households; hence, the findings could be generalizable to the general population in the area. Limitations include the following: children with asymptomatic and symptomatic malaria were not assessed separately. Community-level clustering was not accounted for when performing statistics. Highly sensitive polymerase chain reaction (PCR) was not applied for *Plasmodium* species detection. Another limitation was related to the nature of the cross-sectional study design, which does not confirm a definitive cause-and-effect relationship. Response bias may be introduced during face-to-face interviews.

## Conclusion

The current study shows that the prevalence of malaria among children under 5 years old was high in this pastoral region of Ethiopia. The predominant diagnosed *Plasmodium* species was *P. falciparum* (68.4%). Factors such as child age groups, child fever history, and sleeping under ITN were significantly associated with malaria. The study revealed that households of the pastoral community had low ITN use. To stop the spread of malaria, the local government and other concerned bodies should educate the community about malaria prevention activities and focus on the regular use of ITN. Parents should give priority to children to protect against malaria more effectively, and health facilities should provide effective antimalarial treatment to children with fever.

## Data availability statement

The raw data supporting the conclusions of this article will be made available by the authors, without undue reservation.

## Ethics statement

The studies involving humans were approved by Bule Hora University Institutional Review Board (BHU/IRB/2404/14). The studies were conducted in accordance with the local legislation and institutional requirements. Written informed consent for participation in this study was provided by the participants’ legal guardians/next of kin.

## Author contributions

AA: Conceptualization, Data curation, Formal analysis, Funding acquisition, Investigation, Methodology, Software, Validation, Visualization, Writing – original draft, Writing – review & editing. WG: Conceptualization, Formal analysis, Investigation, Methodology, Project administration, Supervision, Visualization, Writing – original draft, Writing – review & editing. AF: Conceptualization, Data curation, Methodology, Resources, Software, Supervision, Validation, Visualization, Writing – original draft, Writing – review & editing.
