# Supplementary material for: Malaria and associated factors among under-five children in Borena pastoral communities, southern Ethiopia
Source: Front Parasitol. 2024 Aug 1;3:1438218. doi: 10.3389/fpara.2024.1438218 (PMC11731693; doi:10.3389/fpara.2024.1438218)
Supplement: Supplementary file 1 [file Table_1.docx]

**Annex I: Questionnaires**

1. Questionnaire ID number___________

2. Name of the Kebele ______________

Question Circle one Date I----------------

**1. Questionnaire**

| **S.No** | **Variable** | **Response** |
| --- | --- | --- |
| **Part I. Sociodemographic characteristics** | | |
| Q101 | How old your child is? | ----------years |
| Q102 | What is the gender of your child?? | 1. Male 2. Female |
| Q103 | What is the educational mothers/guardian? | 1. Unable to read and write  2. Able to read and write  3. Grade 1-8 (primary)  4. Grade 9-12 (Secondary)  5. College and above |
| Q104 | What is the occupation mothers/guardian? | 1. Herder  2. House wife  3. Merchant  4. Farmer  5. Private employee  6. Government employee  7. Other (specify) ________ |
| Q105 | How many under five children in your house? | 1.­­­­­­­­­­­­­­­­­­­­­­­­­­­­­­ One  2. ≥ Two |
| Q106 | Where your family living place? | 1. Urban  2. Rural |
| Q107 | How many your household monthly income? | 1. Low≤1000 ETB  2. Medium=1001–2575 ETB,  3. High >2575 ETB |
| **Part II. Mosquito control practices** | | |
|  | Does spray indoor residual (insecticide) in last one year to your house? | 1. Yes  2. No |
|  | Does child slept under insecticide treated net (ITN) in home? | A. Yes B. No |
|  | Is any livestock present inside the house | A. Yes B. No |
| **Part III. Health conditions** | | |
| Q301 | Is child fever history within past one week? | 1. Yes 2. No |
| Q302 | Is any household previous infected with malaria? | 1. Yes 2. No |

**2. Microscope examination report**

| **S.No.** | **Variables** | **Results** |
| --- | --- | --- |
| L101. | Plasmodium species | 1. Present  2. Absent |
| L102. | If present, types of plasmodium species | 1. *P.falciparum*  2. *P.vivax*  3. Mixed (Both) |

**Annex II: Laboratory procedures**

**Blood collection and malaria microscope examination procedures**

**I*. Capillary blood collection***

1. Label precleaned slides with the participant’s code

2. Clean the site well with alcohol

3. Allow to air dry

4. Prick the Palmer surface of the tip of the ring (4th) or middle finger (3rd) or plantar surface of the big toe or the heel (in infants).

5. The first drop of free blood was wiped away with clean gauze.

6. Prepare at least 2 thick smears and 2 thin smears.

**II. *Blood film smear preparation***

**A. Thick blood film**

i. Prepare at least 2 smears per participant!

ii. Place a small drop of blood in the center of the precleaned, 2 labeled slide.

iii. Using the corner of another slide or an applicator stick spread the drop in a circular pattern until it was the size of a dime (1.5 cm^2^).

iv. A thick smear of proper density is one which, if placed (wet) over newsprint, allows you to barely read the words.

v. Lay the slides flat and allow the smears to dry thoroughly (protect from dust and insects).

**B. Thin blood film**

i. Prepare at least 2 smears per patient!

ii. Place a small drop of blood on the precleaned, labeled slide near its frosted end (approximately ¼ inch from the frosted area of the glass slide).

iii. Bring another slide at a 30-45° angle up to the drop, allowing the drop to spread along the contact line of the 2 slides.

iv. Quickly push the upper (spreader) slide toward the unfrosted end of the lower slide.

v. Ensuring that the smears have a good feathered edge.

vi. Allow the thin smears to dry.

vii. The smears were fixed by dipping them in absolute methanol.

**III. *Staining the blood films***

i. The concentrated Giemsa stain was diluted by adding 1 volume of stain to 9 volumes of buffered diluted water.

ii. The fixed BF with 1:10 diluted Giemsa stain was added.

iii. Allow to stand for 10 minutes.

iv. The stain was washed off with water.

v. The water was removed under the side of the slide, wiped free of stain and allowed to air dry.

vi. Examine the film under the microscope

**IV. *Microscopic examination of smears***

i. Examine the smear using the 100× oil immersion objective lens.

ii. An area that is well stained, free of stain precipitate and well-populated with white blood cells (WBCs) (10-20 WBCs/field) was selected.

iii. If you see parasites, make a tentative species determination on the thick smear and then examine the thin smear to determine the species present.

**V. *Reporting BF Results***

I. If positive, indicate the species, stage. Mixed infection

II. If negative, after at least 100x thick fields: No parasite/hemo-parasite found
